# Bilateral transversus abdominis plane (TAP) block with 24 hours ropivacaine infusion via TAP catheters: A randomized trial in healthy volunteers

**DOI:** 10.1186/1471-2253-13-30

**Published:** 2013-10-10

**Authors:** Pernille L Petersen, Karen L Hilsted, Jørgen B Dahl, Ole Mathiesen

**Affiliations:** 1 Department of Anesthesia, Centre of Head and Orthopedics, Copenhagen University Hospital, Rigshospitalet, Copenhagen, Denmark; 2 Section of Acute Pain Management, Copenhagen University Hospital, Rigshospitalet, Copenhagen, Denmark

**Keywords:** Transversus abdominis plane block, Transversus abdominis plane catheters

## Abstract

**Background:**

The analgesic effect of a TAP block has been investigated in various surgical settings. There are however limited information about block level and block duration. Furthermore, there is a lack of information about continuous TAP block after ultrasound-guided posterior TAP blocks.

The aim of this double-blind randomized study was therefore to investigate the effect of an ultrasound-guided posterior TAP block with 24 hours local anesthetic infusion via a TAP catheter.

**Methods:**

In this randomized study 8 male volunteers received a bilateral TAP block (20 mLs 0.5% ropivacaine) and were allocated to receive active infusion (ropivacaine 0.2% 5 mL/hr) via a TAP catheter on one side and placebo infusion on the other side. Primary outcome: Dermatomal sensory block involvement after 24 hours evaluated with pinprick. Secondary outcomes: Sensory block involvement evaluated with cold test and heat-pain detection thresholds (HPDT) on the abdominal wall. Assessment points: 15 min before block performance and 1, 4, 8, 12 and 24 hours after block performance.

**Results:**

The TAP block primarily involved sensory changes in the Th10 to Th12 dermatomes. On the placebo side there was a decrease in extension beginning at 4–8 hours after block performance and with no detectable effect beyond 12 hours. Median number of dermatomes anesthetized (pinprick) at 24 hours after block performance was 1.5 (0–3) on the active side compared with 0 (0–0) on the placebo side (P = 0.039).

There were no statistical significant between-side differences in HPDT measurements at 24 hours after block performance.

**Conclusions:**

The spread of sensory block following ultrasound-guided posterior TAP block is partly maintained by a continuous 24 hour ropivacaine infusion through a TAP catheter.

**Trial registration:**

The study was registered at NCT01577940

## Background

Transversus abdominis plane (TAP) block is a regional anesthetic technique that blocks neural afferents of the anterolateral abdominal wall. Nerves located within the TAP are the intercostal, subcostal and ilioinguinal/iliohypogastric nerves (T6-L1).

The analgesic effect of a TAP block has been investigated in various surgical settings. There are however limited information about block level and block duration. Furthermore, there is a lack of information about continuous TAP block after ultrasound-guided posterior TAP blocks [1]. Continuous peripheral nerve blocks provide superior postoperative analgesia when compared with opioid analgesia [2]. Hebbard et al. described a technique for continuous oblique subcostal TAP block, where infusion with ropivacaine 0.2% 5 mL/hr via subcostal TAP catheters was used [3].

The aim of this double-blind randomized study was to investigate the effect of an ultrasound- guided posterior TAP block with 24 hours local anesthetic infusion via a TAP catheter. The primary outcome was dermatomal sensory block involvement after 24 hours evaluated with pinprick. Secondary endpoints were dermatomal sensory block involvement evaluated with cold test and sensory block evaluation with Heat Pain Detection Threshold (HPDT) measurements.

## Methods

The study was carried out at Rigshospitalet, Copenhagen, Denmark. Approval was obtained from the Regional Ethics Committee, the Danish Medicine Agency, and the Danish Data Protection Agency. The study was conducted in compliance with guidelines for Good Clinical Practice (GCP) and was monitored by The Copenhagen University Hospital GCP unit. Furthermore, the design and the description of the study are in accordance with the Consolidated Standards of Reporting Clinical Trials (CONSORT) [4]. The study was registered at http://www.clinicaltrials.gov in December 2011.

### Participants

Male volunteers (18–30 years) with American Society of Anesthesiology performance status 1 were included in the study. Exclusion criteria were as follows: BMI below 18 or above 25 kg/m^2^, inability to understand Danish, relevant drug allergy, alcohol or drug abuse, a daily intake of prescription pain medication for the last 4 weeks, consumption of pain medications within 48 hours before study inclusion and previous abdominal operations.

The volunteers received both written and oral information regarding the trial. Signed informed consent was obtained from all volunteers.

### Interventions, blinding and randomization

This was a randomized, double-blinded and placebo controlled study. An US-guided TAP block was performed bilaterally in all volunteers by one investigator (PLP). An ultrasound probe (GE Health care, Venue 40) was placed transversely in the midaxillary line between the iliac crest and the costal margin at the level of the umbilicus. The external oblique, internal oblique and transversus abdominis muscles and their fascia were visualized. An 18-gauge 100 mm needle (Contiplex S ultraset, B. Braun Medical A/S) was introduced anteriorly and in the plane of the US-probe. Following negative aspiration, 20 mL of ropivacaine 0.5% was injected into the TAP and the injectate was seen spreading in the transversus abdominis plane as a dark oval shape. A TAP-catheter was placed in the TAP fascial space after bolus injection. An infusion pump (Coopdech Syrinjector, Daiken Medical) with 120 mL of study solution was connected to the TAP catheter and a volume of 5 mL/hour was infused via the catheter.

The volunteers were randomly assigned to one of 2 groups. Group 1: Ropivacaine 0.2% in the TAP catheter on the left side and saline in the catheter on the right side. Group 2: Ropivacaine 0.2% in the TAP catheter on the right side and saline on the left side. Study medication was prepared by the hospital pharmacy into identical boxes containing identical ampoules of either 6 × 20 mL of isotonic saline or 6 × 20 mL of 0.2% ropivacaine, with two boxes for each patient marked left and right. The boxes were sealed and marked with the name of the project, the investigators name, and consecutive numbers according to a computer-generated block randomization list prepared by the hospital pharmacy. The volunteers and the investigators were blinded to group assignments. The investigators (PLP, KLH) performed all assessments. The volunteers stayed in hospital for 24 hours followed by a phone call at home after 48 hours.

### Outcomes

The participants were assessed 15 minutes before block performance and at 1, 4, 8, 12 and 24 hours after TAP block performance.

The primary outcome measure of the study was difference in extend of sensory block at 24 hours post block between groups estimated with pinprick on the abdominal skin 5 cm lateral of the midline.

The secondary outcomes measures were spread and extend of sensory block estimated with cold test and Heat Pain Detection Threshold (HPDT) on the abdominal wall bilaterally.

HPDT represents the lowest temperature that is perceived as painful. A thermode (12.5 cm^2^, Thermotest, Somedic A/B, Hörby, Sweden) was placed on normal skin of the abdominal wall, 5 cm below the umbilicus and 5 cm lateral to the midline bilaterally. The starting temperature of the thermode was 32°C, and the rate of increase was 1°C/s. By pressing a button, subjects indicated when the threshold was reached. If the cutoff limit (52°C) was reached before the pertinent threshold, the thermode returned automatically to the starting value, and 52°C was registered. Each threshold was calculated as an average of four stimulations; stimulations were 6–10 s apart.

### Sample size

We were not able to find any study that demonstrated the sensory spread after 24-hour infusion via TAP catheters. In a previous study the mean sensory extend of a bilateral TAP block with 2 × 20 mL of ropivacaine were 6 dermatomes (SD 1.7) for pinprick evaluated 30 minutes after block performance [5]. Based on the sensory spread at 30 minutes after block performance, we thought that a difference of 30% of dermatomes involved (= 2 dermatomes) between the active and the placebo infusion side after 24 hours infusion via TAP catheters was estimated to be of clinical relevance. With a Type 1 error of 0.05 and a Type 2 error of 0.20 a sample size calculation determined that 8 volunteers were needed in the study.

### Statistical methods

Statistical analyses were performed using SPSS 19 (SPSS, Chicago, Illinois, USA). Data are presented as median and range (minimum-maximum). The Wilcoxon signed rank test was used to test for differences between groups at 24 hours post-block. The investigators did all statistical analyses.

P< 0.05 was considered as level of significance.

## Results

Eight volunteers were approached for participation in the study in January 2012. All 8 were recruited and randomly assigned to their treatment group. Baseline characteristics were as follows: Median age 23 years (20–26), height 180 cm (177–198) and weight 80 kg (71–93).

**Figure 1:**
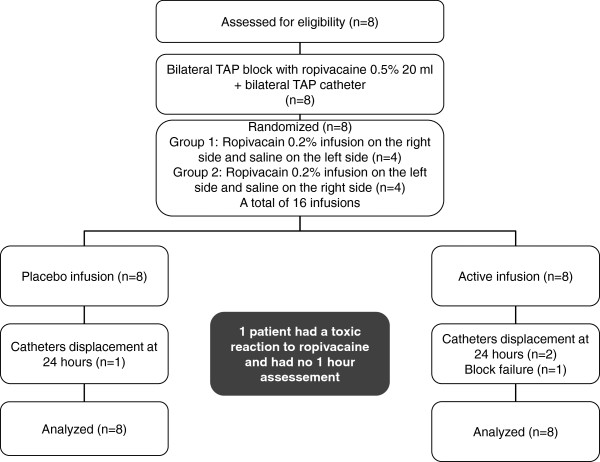
**Flow diagram over volunteer distribution.** Flow diagram over volunteer inclusion, randomization and interventions.

A total of 16 TAP blocks and TAP catheters were performed. All blocks were performed by one investigator (PLP) as described in the methods section. Three out of the 16 catheters were displaced between 12 and 24 hours after block performance and 1 block failed to produce any sensory block at all time points assessed. Results from all 16 infusions were included in the final intention-to-treat analysis (Figure 1).

We suspected a toxic reaction of ropivacaine in one participant 1 hour after block performance. However, complete recovery was achieved within 1 hour after infusion of 2000 mL of Ringers lactate. This volunteer was not assessed at 1 hour post block but data from all other time-points are included.

### Primary outcome

The mean number of dermatomes anesthetized at 24 hours post block evaluated with pinprick was 1.5 (0–3) on the active side compared with 0 (0–0) on the placebo side (P = 0.039).

The median upper and lower dermatome involvement is presented in Table 1.

**Table 1:** Dermatomes involved in TAP blockade

	**Active**	**Placebo**	
Number of dermatomes involved at 24 h			
Pin Prick	1.5 (0–3)	0 (0–0)	P=0.039
Heat/Cold	2.0 (0–3)	0 (0–0)	P=0.034
Upper and lower dermatomal involvement (Pin Prick)			
1h	T10 to T12	T10 to T12	
4h	T10 to T11	T10 to T12	
8h	T11 to T12	T11 to T12	
12h	T10 to T12	None	
24h	T10 to T11	None	
Upper and lower dermatomal involvement (cold test)			
1h	T10 to T12	T10 to T12	
4h	T10 to T12	T10 to T12	
8h	T11 to T12	T10 to T12	
12h	T11 to T12	None	
24h	T10 to T11	None	

Median and (minimum-maximum).

Sensory spread after TAP block and infusion via TAP catheters. Active indicates infusion with ropivacaine and placebo with saline in the TAP catheters. The hours indicate time from TAP block performance.

### Secondary outcomes

#### Extend of sensory block estimated with cold test

The mean number of dermatomes anesthetized at 24 hours evaluated with cold test were 2 (0–3) on the active side and 0 (0–0) on the placebo side (P=0.034).

#### Sensory block estimated with heat pain detection threshold (HPDT)

**Figure 2:**
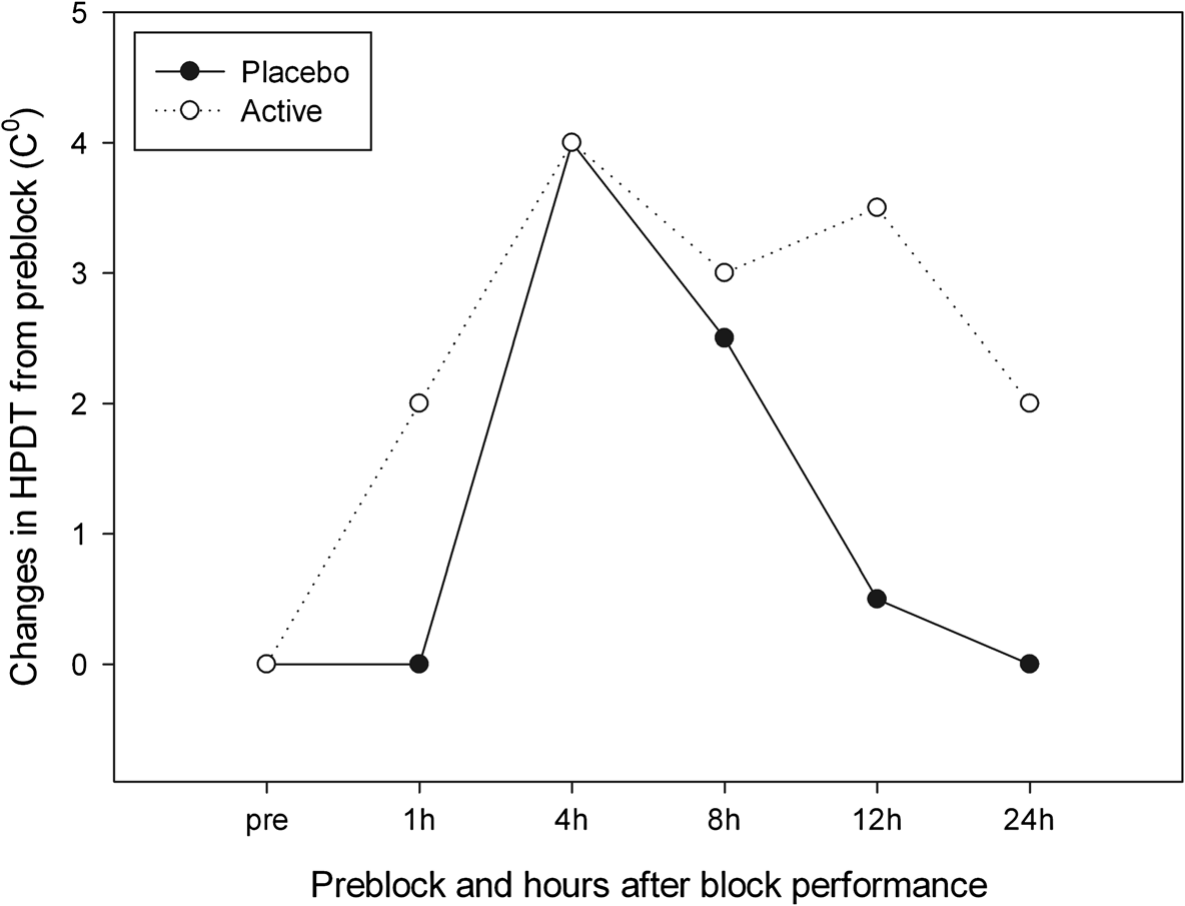
**Heat pain detection threshold (HPDT).** HPDT represents the lowest temperature that is perceived as painful. Measurements were conducted on abdominal skin bilaterally before block performance and 1, 4, 8, 12 and 24 hours after block performance.

Levels of HPDT (abdomen) scores were not significantly different between groups at 24 hours. HPDT score at each time point assessed are presented in Figure 2. The figure shows a maximum effect of the TAP block at 4h after block performance which is partly maintained on the active side at 24 hours and ceased on the placebo side.

## Discussion

This randomized controlled trial showed that both a pinprick and a cold test sensory block produced with a TAP block can be maintained with an infusion of local anesthetics via a TAP catheter. We have demonstrated a significant difference between placebo and active infusion for our primary endpoint, dermatomal involvement at 24 hours after TAP block performance. Furthermore, we believe that the differences in HPDT scores between the two groups supports this primary result, although these scores failed to display significance at 24 hours after block performance. Due to the limited sensory spread of the TAP block, the thermode that was used for the HPDT measurements was unavoidably placed partly on non anesthetized skin which may have interfered with assessments.

The TAP block primarily involved sensory changes in the Th10 to Th12 dermatomes. On the placebo side there was a decrease in extension beginning at 4–8 hours post block and with no detectable effect beyond 12 hours. In another recent study with healthy volunteers the dermatomal involvement after posterior TAP blockade was comparable to our study [6]. However; in this study 30 mL of ropivacaine 0.375% was used for the block. In a different study Lee et al. [7] demonstrated a primary TAP block involvement of Th10 to Th12 in patients undergoing different types of abdominal surgery, but also found involvement of T9 in 30% and L1 in 50% of the TAP blocks which even lasted for up to 24 hours in some cases. Finally, Mitchell et al. [5] demonstrated a sensory dermatomal involvement of primarily Th10 –Th12 in a group of abdominal surgery patients. These patients all received a posterior TAP block (2 × 20 mL of ropivacaine 0.5%) preoperatively and were tested for spread for the following 30 minutes. Collectively, data indicate that in both patients and volunteers, an ultrasound guided posterior TAP block only produces a limited sensory block.

In this study we suspected a toxic reaction to local anesthetic 1 hour after TAP block performance in one participant. Three studies have demonstrated a risk of systemic toxicity of local anesthetic as a result of absorption into the circulation after TAP block performance with both lidocaine and ropivacaine [8-10]. These studies found a mean peak plasma concentration 30 minutes after block performance. Griffiths found a level of plasma ropivacaine over the toxic level that was maintained for several hours in some patients [9]. A recent study demonstrated reduced peak plasma concentrations of local anesthetics after unilateral TAP block when epinephrine was added to the solution [11]. Absorption of the local anesthetic into the circulation depends primarily on the vascularity of the site of deposition [12]. As the TAP is well vascularised and the area of absorption rather large it can explain the high peak plasma concentrations of local anesthetics in this type of block.

There are some limitations to our study. Three of sixteen catheters were displaced from 12 to 24 hours and we experienced one block failure. Niraj et al. studied the analgesic effect of subcostal transversus abdominis plane catheters versus epidural catheters, and in that study 45% of TAP catheters had to be re-sited within the first 24 hours after surgery [13]. In a study of pulmonary function in healthy volunteers that had bilateral TAP blockade, 2 out of 11 volunteers had bilateral block failure [14]. We should have anticipated problems with block failure and catheter displacements and taken this into account in our sample size calculation. However, we found a statistically significant difference in our primary outcome despite of block failures and catheter displacement. Another limitation is that the study was conducted on healthy volunteers, the insertion of TAP catheters and their likely success is more problematic in a surgical population.

## Conclusions

This study demonstrates that an ultrasound-guided posterior TAP block produces a limited sensory spread with primary involvement of Th10-Th12 in healthy male volunteers. Furthermore, the spread of sensory block are partly maintained by a continuous 24 hour ropivacaine infusion via a TAP catheter.

## Competing interests

The authors declare no competing interests.

## Authors’ contributions

PLP, JBD and OM designed the experiments. PLP and KLH collected the data. PLP, JBD and OM analysed the data and drafted the manuscript. All authors read and approved the final manuscript.

## Pre-publication history

The pre-publication history for this paper can be accessed here:

http://www.biomedcentral.com/1471-2253/13/30/prepub
